# Binge eating, trauma, and suicide attempt in community adults with major depressive disorder

**DOI:** 10.1371/journal.pone.0198192

**Published:** 2018-06-21

**Authors:** Ji Hyun Baek, Kiwon Kim, Jin Pyo Hong, Maeng Je Cho, Maurizio Fava, David Mischoulon, Sung Man Chang, Ji Yeon Kim, Hana Cho, Hong Jin Jeon

**Affiliations:** 1 Department of Psychiatry, Depression Center, Samsung Medical Center, Sungkyunkwan University School of Medicine, Seoul, South Korea; 2 Department of Psychiatry & Behavioral Science, Seoul National University College of Medicine, Seoul, South Korea; 3 Depression Clinical and Research Program, Massachusetts General Hospital, Harvard Medical School, Boston, United States of America; 4 Department of Psychiatry, School of Medicine, Kyungpook National University, Daegu, South Korea; 5 Department of Physiology, Sungkyunkwan University School of Medicine, Samsung Biomedical Research Institute, Suwon, Korea; 6 Department of Health Sciences & Technology, Department of Medical Device Management & Research, and Department of Clinical Research Design & Evaluation, Samsung Advanced Institute for Health Sciences & Technology (SAIHST), Sungkyunkwan University, Seoul, Korea; Public Library of Science, UNITED STATES

## Abstract

Eating disorders comorbid with depression are an established risk factor for suicide. In this study, we aimed to determine the effects of binge eating (BE) symptoms on suicidality and related clinical characteristics in major depressive disorder (MDD). A total of 817 community participants with MDD were included. We compared two groups (with and without lifetime BE symptoms). The MDD with BE group was subdivided into a frequent BE (FBE) subgroup (BE symptoms greater than twice weekly) and any BE (ABE) subgroup (BE symptoms greater than twice weekly). The MDD with BE group comprised 142 (17.38%) patients. The FBE and ABE subgroups comprised 75 (9.18%) and 67 (8.20%) patients, respectively. Comorbid alcohol use disorder, anxiety disorder, post-traumatic stress disorder (PTSD) and history of suicide attempt were significantly more frequent in the MDD with BE group than MDD without BE group. Sexual trauma was also reported more frequently in MDD with BE group. No significant differences were observed between the ABE and FBE subgroups. Multivariate logistic regression revealed an association of suicide attempt with BE symptoms and sexual trauma. Structural equation modeling showed that sexual trauma increased BE (β = 0.337, *P* <0.001) together with alcohol use (β = 0.185, *P* <0.001) and anxiety (β = 0.299, p<0.001), which in turn increased suicide attempt (β = 0.087, p = 0.011). BE symptoms were associated with suicide attempt in MDD after adjusting for other factors associated with suicidality. BE symptoms also moderated an association between suicide attempt and sexual trauma.

## Introduction

Depression is one of the strongest risk factors for suicide. However, which aspects of depression specifically increase suicide risk remain unclear. Depression is a heterogeneous condition with various clinical characteristics that can independently increase suicide risk. Comorbid psychiatric conditions may also contribute to suicide risk [1–3]. In addition, co-existing psychiatric symptoms that do not meet the full Diagnostic and Statistical Manual of Mental Disorders (DSM) criteria for comorbid conditions may also increase suicide risk.

Disordered eating symptoms are among the widely reported and easily observable psychiatric symptoms in depression. Emotional eating [4] is a well-known phenomenon frequently observed in depression. Binge eating (BE) symptoms are associated with depression and impulsivity [5]. Eating disorders including anorexia nervosa, bulimia nervosa, and BE disorder are known risk factors for suicide [6, 7]. However, it is unclear if individual disordered eating symptoms, especially those of BE, are also individually associated with suicidality in MDD.

In this study, we used an epidemiological data set to determine the possible association of dysfunctional eating behavior (i.e., BE behavior) with clinical characteristics and suicidality in patients with MDD. We hypothesized that the presence of BE symptoms would be associated with greater frequency of suicidality.

## Materials and methods

### Data sources, data collection, and study sample

Data were extracted from a nationwide epidemiological study of Korean adults, the Korean Epidemiologic Catchment Area Study Replication (KECA-R) and Korean Epidemiologic Catchment Area Study 2011 (KECA-2011). Details about the study procedure are described elsewhere [8–10]. In brief, KECA aimed to estimate the prevalence of the DSM-IV psychiatric disorders in the Korean population. Subjects were selected by a multi-stage, cluster sampling of 7,867 adult household residents in 12 catchment areas. One adult who was 18 years or older per selected household was chosen at random, and face-to-face interviews were conducted using the Korean version of Composite International Diagnostic Interview (K-CIDI) [11]. KECA-R was conducted from July 2006 to April 2007 and KECA-2011 was conducted from July to September 2011. Both studies had independent study samples with the same study design. A total of 12,532 adults were included in the two cohorts (overall response rate 80.2%). The institutional review board of Seoul National University College of Medicine approved this study. All subjects were fully informed about the aims and methods of the study prior to completing the interview, and informed consent was obtained prior to participation. Of all subjects, 823 were assessed as having a lifetime history of MDD. These individuals were included in the present study.

### Measures

#### Assessment of DSM-IV disorders

DSM-IV diagnoses were based on the K-CIDI [12], a fully structured diagnostic interview designed to make psychiatric diagnoses [13]. K-CIDI has been validated [11] according to World Health Organization guidelines [14]. Furthermore, clinical diagnosis with blind clinical re-interviews, using the Structured Clinical Interview for the DSM-IV (SCID), showed modest concordance with the K-CIDI diagnoses (κ values between 0.50 and 1.00) [15]. Depression symptomatology and psychiatric comorbid conditions were evaluated using the results of the K-CIDI interviews.

#### Assessment of BE symptoms

The eating disorder section of the K-CIDI was used to evaluate lifetime history of BE symptoms independent from depression. Since our aim was to determine the effect of BE symptoms that did not meet the full DSM criteria for eating disorder on suicidality, we excluded those who met the criteria for eating disorder (n = 6) in the analyses. Those who reported BE symptoms more than twice a week were classified in the frequent binge eating (FBE) group. Those who reported experiencing BE symptoms less frequently were classified in the any binge eating (ABE) group. In evaluating BE, interviewers explicitly asked using following question: “Have you ever eaten abnormally large amounts of food within a short period of time? In other words, have you ever engaged in BE?” If the response was yes, the interviewers inquired further about the types of food and the amounts eaten. Only in cases where the types and amount of food met the general concept of BE symptoms was the response coded as ‘yes’; these individuals comprised the MDD with BE group. Subjects who had responded ‘yes’ were also asked the question: “Have you ever experienced such kind of BE more than twice per week?” Those who said yes to this question were classified in the FBE group.

#### Assessment of lifetime suicide attempt

Interviewers asked all subjects about suicidal ideation, plans, and attempts in a standard and consistent way [12,14] using the following explicit questions:

- “Have you ever seriously thought about committing suicide?”, for suicidal ideation
- “Have you ever made a plan for committing suicide?”, for suicidal planning
- “Have you ever attempted suicide?”, for suicide attempt

The participants responded ‘yes’ or ‘no’; those responding ‘yes’ were asked their age at the first suicide attempt and the number of lifetime suicide attempts. The questions showed strong validity between psychiatrists and interviewers of this study, as well as inter-rater and test-retest reliability with kappa values between 0.74 and 1.00 and 0.84 for MDD in a preliminary study for the KECA-R [16].

### Statistical analyses

Considering the exploratory nature of this study, statistical significance was evaluated using a two-sided design with alpha set a priori at 0.01. Chi-square test was performed to compare rate of categorical variables between MDD with and without BE symptoms. Analyses of covariance (ANCOVA) were conducted to compare continuous variables between two after controlling for age, sex, and income. Multinomial logistic regression models were used to compare the detailed depression symptomatology and clinical characteristics between three groups. Age, sex, and income were applied as covariates. We additionally performed logistic regression analyses to assess whether BE symptom was associated with elevated risk of lifetime suicide attempt after adjusting for all other co-occurring clinical characteristics. All analyses were performed with SPSS 24.0 (SPSS, Chicago, IL).

## Results

Among 823 subjects with MDD, comorbid eating disorder was reported in six, who were excluded, leaving 817 subjects. Among them, 142 (17.38%) reported experiencing at least one BE symptom. Among these, 75 (8.20%) reported FBE symptoms, with the remaining 67 (9.18%) reporting ABE symptoms. Table 1 provides the sociodemographic characteristics of the subjects. The MDD wit BE group were younger than the MDD without BE group. The age at onset of depression was younger in the MDD with BE group.

**Table 1:** Sociodemographic profiles of the 817 subjects.

Variables	MDD without BE (N = 675)	MDD with BE (n = 142)		Statistics	
		MDD with ABE (N = 67)	MDD with FBE (N = 75)		
	Mean (SD)	Mean (SD)	Mean (SD)	F	p-value
**Age (years)**	46.53 (14.01)	37.07 (11.87)	37.93 (13.03)	25.19	<0.001
**Age of onset of MDD (years)**	37.15 (13.27)	29.82 (11.85)	29.49 (11.13)	19.56	<0.001
**Age groups (years)**	% (n)	% (n)	% (n)	χ^2^	*p*-value
≤29	12.4 (84)	34.3 (23)	30.7 (23)	55.33	<0.001
30–39	21.2 (143)	26.9 (18)	28.0 (21)		
40–49	23.9 (161)	23.9 (16)	14.7 (11)		
50–59	22.5 (152)	10.4 (7)	22.7 (17)		
≥ 60	20.0 (135)	4.5 (3)	4.0 (3)		
**Gender**					
Male	22.5 (152)	32.8 (22)	18.7 (14)	4.54	0.103
Female	77.5 (523)	67.2 (45)	81.3 (61)		
**Education (years)**					
11≤	35.4 (239)	23.9 (16)	33.3 (25)	5.52	0.238
12	31.0 (209)	31.3 (21)	26.7 (20)		
12<	33.6 (227)	44.8 (30)	40.0 (30)		
**Employment**					
Full time	64.4 (435)	56.7 (38)	53.3 (40)	6.63	0.157
Part time	6.4 (43)	11.9 (8)	8.0 (6)		
Unemployed	29.2 (197)	31.3 (21)	38.7 (29)		
**Marital status**					
Married	58.5 (395)	49.3 (33)	53.3 (40)	38.99	<0.001
Widowed/divorced/separated	26.4 (178)	10.4 (7)	14.7 (11)		
Unmarried	15.1 (102)	40.3 (27)	32.0 (24)		
**Household income**					
≤2000$ /month	55.9 (314)	43.6 (24)	53.8 (35)	8.89	0.064
2000–3000$ /month	20.5 (115)	14.5 (8)	21.5 (14)		
> 3000$ /month	23.7 (133)	41.8 (23)	24.6 (16)		
**Living area**					
Cities	79.1 (534)	85.1 (57)	84.0 (63)	2.16	0.339
Rural area	20.9 (141)	14.9 (10)	16.0 (12)		

MDD, major depressive disorder; BE, binge eating; ABE, any binge eating; FBE, frequent binge eating. ABE was a lifetime history of BE that does not meet the criteria for FBE. FBE was defined as BE more than twice per week.

### Depressive symptomatology and psychiatric comorbid conditions associated with BE symptoms

Table 2 presents the differences in depressive symptomatology and clinical characteristics between the MDD with FBE, MDD with ABE, and MDD without BE groups. The MDD with FBE group reported increased appetite and weight gain more frequently than the MDD without BE group during depressive episodes. No other significant differences were observed in other depression symptomatology, including hypersomnia or psychomotor symptoms (i.e., psychomotor agitation and retardation). As for comorbid psychiatric conditions, the MDD with ABE and MDD with FBE groups both reported anxiety disorders more frequently. The MDD with FBE group reported more frequent post-traumatic stress disorder (PTSD) than other groups. Significant differences were observed in terms of rate of suicide attempt history between three groups; the MDD with ABE group had higher rates of suicide attempts compared to MDD without BE group (post-hoc *P* = 0.001), and the MDD with FBE showed a trend of experiencing suicide attempts more frequently compared to MDD without BE group (post-hoc *P* = 0.037).

**Table 2:** Comparison of depression symptoms, comorbid psychiatric conditions and suicide-related behavior between MDD with any binge eating (ABE), frequent binge eating (FBE), and without binge eating (BE) groups.

Variables	MDD without BE^1^ (N = 675)	MDD with BE (n = 142)		*P*	Post-hoc analyses								
		MDD with ABE^2^ (N = 67)	MDD with FBE^3^ (N = 75)		^1^ vs. ^2^			^1^ vs. ^3^			^2^ vs. ^3^		
					*P*	AOR	95% CI	*P*	AOR	95% CI	*P*	AOR	95% CI
**Depression symptoms**													
Depressed mood	623 (92.3)	57 (85.1)	71 (94.7)	0.117	0.090	0.49	0.22–1.12	0.344	1.80	0.53–6.12	0.060	0.27	0.07–1.06
Loss of interest	560 (83.0)	59 (88.1)	72 (96.0)	0.055	0.919	1.04	0.47–2.34	0.016	4.32	1.31–14.16	0.048	0.25	0.06–0.99
Fatigue	623 (92.3)	59 (88.1)	72 (96.0)	0.356	0.421	0.68	0.27–1.74	0.267	2.29	0.53–9.91	0.173	0.32	0.06–1.66
Loss of appetite	566 (83.9)	55 (82.1)	55 (73.3)	0.091	0.447	0.75	0.35–1.58	0.032	0.50	0.27–0.94	0.336	1.56	0.63–3.84
Loss of weight	300 (44.4)	35 (52.2)	35 (46.7)	0.779	0.480	1.23	0.70–2.17	0.938	1.02	0.60–1.73	0.590	1.22	0.59–2.52
Increase appetite	64 (9.5)	15 (22.4)	26 (34.7)	**<0.001**	0.016	2.43	1.18–5.01	**<0.001**	**4.70**	**2.58–8.58**	0.104	0.50	0.22–0.84
Weight gain	51 (7.6)	7 (10.4)	23 (30.7)	**<0.001**	0.342	1.53	0.64–3.67	**<0.001**	**4.74**	**2.52–8.92**	0.021	0.32	0.12–0.84
Insomnia	567 (84.0)	58 (86.6)	62 (82.7)	0.238	0.137	1.98	0.81–5.87	0.335	1.44	0.69–2.99	0.491	1.47	0.49–4.41
Hypersomnia	145 (21.5)	19 (28.4)	24 (32.0)	0.719	0.933	1.03	0.54–1.97	0.417	1.27	0.71–2.27	0.608	0.81	0.36–1.81
Psychomotor retardation	391 (58.0)	41 (61.2)	50 (66.7)	0.688	0.721	1.11	0.62–2.00	0.410	1.26	0.73–2.19	0.750	0.89	0.42–1.88
Psychomotor agitation	314 (46.5)	32 (47.8)	42 (56.0)	0.151	0.350	1.32	0.74–2.34	0.071	1.64	0.96–2.79	0.580	0.81	0.39–1.69
Worthlessness	411 (60.9)	41 (61.2)	43 (57.3)	0.765	0.813	1.07	0.60–1.92	0.510	0.84	0.49–1.43	0.470	1.31	0.63–2.74
Feelings of inferiority	468 (69.3)	49 (73.1)	51 (68.0)	0.853	0.916	0.97	0.52–1.80	0.573	0.85	0.48–1.50	0.761	1.13	0.63–2.74
Loss of confidence	394 (58.4)	44 (65.7)	48 (64.0)	0.556	0.398	1.29	0.72–2.32	0.439	1.24	0.72–2.14	0.996	1.00	0.47–2.12
Concentration difficulty	515 (76.3)	57 (85.1)	61 (81.3)	0.322	0.205	1.63	0.77–3.48	0.347	1.38	0.71–2.69	0.749	1.17	0.45–3.04
Feelings of confusion	410 (60.7)	44 (65.7)	51 (68.0)	0.280	0.168	1.53	0.84–2.81	0.339	1.31	0.75–2.29	0.690	1.17	0.54–2.53
Indecisiveness	452 (67.0)	46 (68.7)	48 (64.0)	0.388	0.724	0.90	0.49–1.65	0.173	0.68	0.40–1.18	0.476	1.32	0.62–2.84
Morning worsening	294 (43.6)	34 (50.7)	34 (45.3)	0.835	0.549	1.19	0.67–2.10	0.908	1.03	0.61–1.75	0.733	1.13	0.55–2.34
Loss of sexual interest	416 (61.7)	46 (68.7)	44 (58.7)	0.500	0.348	1.35	0.72–2.52	0.553	0.85	0.49–1.46	0.275	1.54	0.71–3.33
Loss of pleasure	512 (76.0)	48 (71.6)	54 (72.0)	0.568	0.287	0.71	0.38–1.33	0.907	0.97	0.53–1.76	0.458	0.74	0.33–1.65
**Comorbid psychiatric conditions**													
Alcohol use disorder	116 (17.2)	27 (40.3)	20 (26.7)	**0.006**	**0.004**	**2.49**	**1.33–4.66**	0.061	1.81	0.97–3.37	0.346	1.47	0.66–3.24
Anxiety disorder	235 (34.9)	34 (50.7)	40 (54.1)	**<0.001**	**0.008**	**2.20**	**1.23–3.94**	**0.001**	**2.48**	**1.44–4.26**	0.769	0.90	0.43–1.87
Panic disorder	27 (4.0)	3 (4.5)	6 (8.0)	0.182	0.521	1.52	0.42–5.45	0.069	2.47	0.93–6.52	0.528	0.63	0.15–2.67
Agoraphobia	18 (2.7)	4 (6.0)	5 (6.7)	0.249	0.220	2.28	0.61–8.56	0.173	2.25	0.70–7.24	0.972	0.97	0.20–4.65
Social phobia	107 (15.9)	15 (22.4)	18 (24.0)	0.429	0.484	1.30	0.63–2.69	0.234	1.46	0.78–2.74	0.816	0.90	0.37–2.18
Nicotine use disorder	69 (10.2)	12 (17.9)	13 (17.3)	0.047	0.217	1.67	0.74–3.80	0.020	2.55	1.16–5.63	0.482	0.69	0.25–1.94
PTSD	52 (7.7)	10 (14.9)	16 (21.3)	**0.004**	0.075	2.25	0.92–5.49	**0.002**	**3.16**	**1.55–6.44**	0.482	0.70	0.25–1.92
GAD	100 (14.9)	13 (19.4)	17 (23.3)	0.104	0.286	1.54	0.70–3.41	0.048	2.01	1.01–4.02	0.638	0.79	0.30–2.09
OCD	17 (2.5)	4 (6.0)	6 (8.0)	0.016	0.190	2.45	0.64–9.33	0.005	4.50	1.58–12.82	0.456	0.57	0.13–2.47
Lifetime suicide behaviors													
Suicide ideation	359 (53.3)	40 (60.6)	47 (62.7)	0.118	0.321	1.34	0.75–2.41	0.055	1.72	0.99–3.00	0.523	0.78	0.37–1.66
Suicide plan	115 (17.1)	15 (23.1)	20 (26.7)	0.025	0.148	1.68	0.83–3.41	0.012	2.16	1.19–3.95	0.603	0.80	0.37–1.66
Suicide attempt	99 (14.8)	21 (31.8)	18 (24.0)	**0.002**	**0.001**	**2.93**	**1.52–5.64**	0.037	1.94	1.04–3.62	0.333	1.50	0.66–3.40

MDD, major depressive disorder; BE, binge eating; ABE, any binge eating; FBE, frequent binge eating; PTSD, Post-traumatic stress disorder; GAD, Generalized anxiety disorder, OCD, Obsessive compulsive disorder; aOR, adjusted odds ratio; CI, confidence interval. FBE was defined as BE more than twice per week. ABE was a lifetime history of BE that does not meet the criteria for FBE. Adjusted for age, gender, income. Bold indicates statistically significant (p<0.01).

### Trauma experience

The MDD with FBE group had PTSD more frequently than the MDD without BE group. Trauma experience has been suggested as a risk factor for obesity [17]. We hypothesized that trauma experience might contribute to experience of BE symptoms. The PTSD section of K-CIDI was used to evaluate lifetime traumatic events and DSM-IV PTSD criteria from A to F. For the beginning of the section, respondents were asked about traumatic events and experiences. They were handed cards on which were listed 11 kinds of trauma including ‘military combat’, ‘life-threatening accidents’, ‘natural disaster’, ‘witnessed someone being killed or seriously injured’, ‘rape’, ‘sexual assault’, ‘badly beaten’, ‘threatened with a weapon’, ‘held captive, kidnapped, tortured and terrorism’, ‘any other extremely stressful or upsetting event’, and ‘learning about trauma to others’. If ‘any other extremely stressful or upsetting event’ and ‘learning about trauma to others’ were related to bereavement, chronic illness, business loss, or marital or family conflict, they were not regarded as traumatic events. If the subjects indicated experiencing any of these traumatic events, the interviewers continued the PTSD section. After adjusting for age, sex, and income, the MDD with BE group reported experiencing any trauma more often compared to the MDD without BE group (Table 3). The MDD with FBE and MDD with ABE groups experienced both early trauma (trauma experienced at <18 years of age) and adult trauma (>18 years of age) more frequently than the MDD without BE group. In particular, the MDD with BE group had more frequent sexual trauma compared to the MDD without BE group.

**Table 3:** Comparison of types of trauma experienced between MDD with any binge eating (ABE), frequent binge eating (FBE), and without binge eating (BE) groups.

Types of trauma	MDD without BE^1^ (N = 675)	MDD with BE (n = 142)		p	Post-hoc analyses								
					^1^ vs. ^2^			^1^ vs. ^3^			^2^ vs. ^3^		
		MDD with ABE^2^ (N = 67)	MDD with FBE^3^ (N = 75)		p	aOR	95% CI	p	aOR	95% CI	p	aOR	95% CI
Any trauma	234 (34.7)	42 (62.7)	44 (58.7)	**<0.001**	**<0.001**	**3.82**	**2.10–6.95**	**<0.001**	**3.09**	**1.80–5.32**	0.587	1.23	0.58–2.59
Military combat	22 (3.3)	1 (1.5)	1 (1.3)	0.844	0.997	-	-	0.560	3.07	0.18–23.79	0.996	-	-
Life threatening conditions	119 (17.7)	19 (28.4)	15 (20.0)	0.072	0.024	2.16	1.11–4.24	0.447	1.32	0.65–2.70	0.253	1.69	0.69–4.18
Natural disaster or fire	43 (6.4)	9 (13.4)	6 (8.0)	0.041	0.012	3.13	1.29–7.58	0.640	1.30	0.43–3.90	0.168	2.48	0.68–9.00
Witness^a^	90 (13.4)	14 (20.9)	17 (22.7)	0.032	0.115	1.83	0.86–3.90	0.020	2.24	1.14–4.42	0.62	0.79	0.31–2.00
Sexual trauma^b^	30 (4.4)	12 (17.9)	16 (21.3)	**<0.001**	**0.003**	**3.76**	**1.59–8.93**	**<0.001**	**5.00**	**2.41–10.35**	0.508	0.73	0.28–1.88
Badly beaten	46 (6.9)	9 (13.4)	10 (13.3)	0.018	0.051	2.47	1.00–6.15	0.015	2.76	1.22–6.27	0.846	0.90	0.30–2.66
Threatened by others^c^	29 (4.3)	6 (9.0)	6 (8.0)	0.405	0.248	1.98	0.62–6.27	0.372	1.68	0.54–5.21	0.778	1.23	0.29–5.27
According to the age at onset of the trauma													
Experienced the trauma at <18 years old	78 (11.6)	16 (24.2)	16 (21.3)	**<0.001**	**<0.001**	**4.40**	**2.07–9.35**	**0.010**	**2.63**	**1.26–5.49**	0.917	0.96	0.40–2.26
Experienced the trauma at >18 years old	156 (23.1)	26 (38.5)	28 (37.4)	**<0.001**	**0.001**	**3.23**	**1.62–6.44**	**<0.001**	**3.17**	**1.73–5.82**	0.341	1.59	0.61–4.13

MDD, major depressive disorder; BE, binge eating; ABE, any binge eating; FBE, frequent binge eating; aOR, adjusted odds ratio; CI, confidence interval. Adjusted for age, gender, years of education, monthly income, and every other traumas. FBE was defined as BE more than twice per week; ABE was a lifetime history of BE that does not meet the criteria for FBE; Bold indicates statistically significant (p<0.01).

^a^ Witness someone being killed or seriously injured

^b^ Rape or sexual assault

^c^ Threatened with a weapon, held captive, kidnapped, tortured, or terrorized

### Suicidality and sexual trauma, BE symptoms

Logistic regression analyses were carried out to evaluate the role of BE symptoms on experience of suicide attempt (Table 4). Considering sexual trauma was associated with both suicide attempt and BE symptoms, we added an interaction term between BE symptoms and sexual trauma experience as a covariate as well as comorbid anxiety disorder and alcohol use disorders. When the group memberships were entered as independent variables, model χ^2^ was 61.66 (df = 11, *P* <0.001). Using this model, 83.3% of the subjects were classified correctly with regard to suicide attempts. Comorbid anxiety disorder and comorbid alcohol use disorder were significantly associated with suicide attempt. BE symptoms and sexual trauma were associated with suicide attempts although the association did not reach statistical significance (BE: p = 0.028; ABE Odds Ratio [OR] 2.68, 95% Confidence Interval [CI] = 1.29–5.58, *P* = 0.008; FBE symptom OR = 1.40, 95% CI-0.64–3.07, *P* = 0.397; sexual trauma: OR = 3.06, 95% CI = 1.27–7.41, *P* = 0.013). We also performed the binary logistic regression analysis using MDD with and without BE group memberships as independent variables. Model χ^2^ was 59.95 (df = 9, *P* <0.001). Using this model, 84.3% of the subjects were classified correctly. Similar to the prior logistic regression model, sexual trauma and BE symptoms showed associations with the suicide attempt without reaching the statistical significance (BE symptom: OR 1.95, 95% CI = 1.10–3.45, p = 0.022; sexual trauma: OR 3.06, 95% CI = 1.27–7.41, *P* = 0.013).

**Table 4:** Logistic regression analyses to determine factors associated with suicide attempt.

	β	SE	OR	95% CI	p
**Model 1: MDD with and without BE as independent variables**					
Age	-0.007	0.009	0.99	0.98–1.01	0.426
Sex (female)	0.446	0.281	1.56	0.90–2.71	0.112
Income					0.025
- Middle income ($ 2000-3000/ month)	-0.242	0.303	0.79	0.43–1.42	0.785
- High income (>$3000/ month)	-0.831	0.305	0.44	0.24–0.79	0.007
Comorbid anxiety disorder	0.850	0.224	2.34	1.51–3.63	<0.001
Comorbid alcohol use disorder	0.782	0.257	2.19	1.32–3.62	0.002
BE symptom	0.666	0.291	1.95	1.10–3.45	0.022
Sexual trauma	1.119	0.451	3.06	1.27–7.41	0.013
Interaction between BE symptom and sexual trauma	-1.071	0.687	0.34	0.09–1.32	0.119
**Model 2: MDD with ABE, FBE and without BE as independent variables**					
Age	-0.007	0.009	0.99	0.98–1.01	0.433
Sex (female)	0.467	0.283	1.60	0.92–2.78	0.099
Income					0.023
- Middle income ($ 2000-3000/ month)	-0.245	0.303	0.78	0.43–1.42	0.418
- High income (>$3000/ month)	-0.848	0.309	0.43	0.23–0.78	0.006
Comorbid anxiety disorder	0.848	0.224	2.34	1.51–3.62	<0.001
Comorbid alcohol use disorder	0.758	0.259	2.14	1.29–3.55	0.003
BE					0.028
- ABE	0.986	0.374	2.680	1.29–5.58	0.008
- FBE	0.338	0.399	1.40	0.64–3.07	0.397
Sexual trauma	1.119	0.451	3.06	1.27–7.41	0.013
Interaction between BE symptom and sexual trauma			0.303		0.303
- Between ABE and sexual trauma	-1.471	1.013	0.23	0.03–1.67	0.146
- Between FBE and sexual trauma	-0.699	0.798	0.50	0.10–2.37	0.381

MDD, major depressive disorder; BE, binge eating; ABE, any binge eating; FBE, frequent binge eating; AOR, adjusted odds ratio; CI, confidence interval. FBE was defined as BE more than twice per week. ABE was a lifetime history of BE that does not meet the criteria for FBE.

### Structural equation modeling (SEM) to determine the causal relationship between sexual trauma, BE symptoms, and suicide attempt

**Fig 1:**
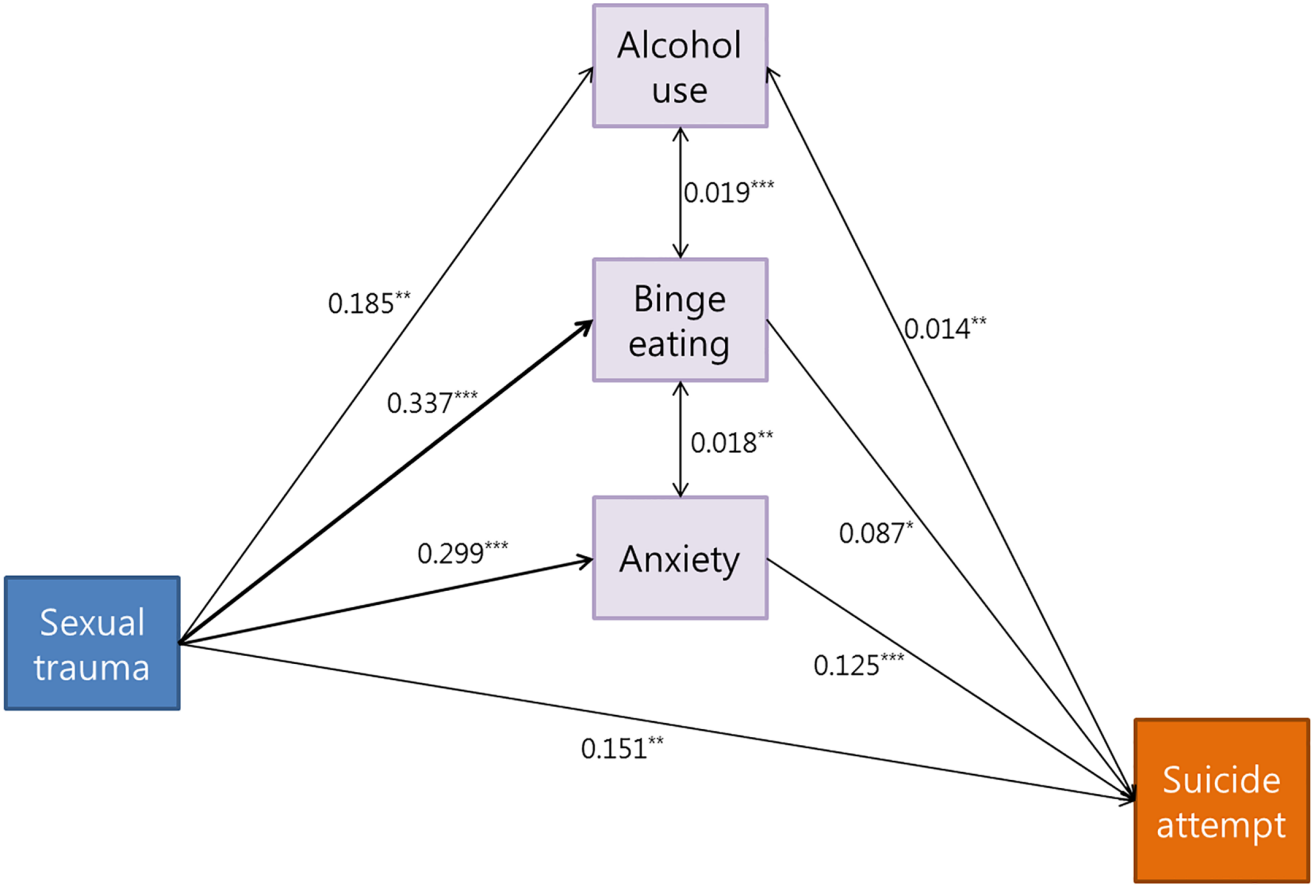
Structural equation modeling among sexual trauma, alcohol use, BE, anxiety, and suicide attempt. All values are standardized regression weights. The thickness of arrow is also determined by the standardized regression weights. *p<0.05, **p<0.01, ***p<0.001.

To infer the relationship between sexual trauma and other variables, we conducted a path analysis, a special case of structural equation modeling that has been popularly used for causal inference in various fields [18, 19]. All analyses were executed by the Lavvan package in R 3.3.2 statistical software [20]. SEM provided good fit to the data (χ^2^ = 5.689, df = 1, *P* = 0.0017, CFI = 0.97, NFI = 0.97, RMSEA = 0.08). In this model, sexual trauma was associated with alcohol use (β = 0.185, *P* <0.001), BE symptoms (β = 0.337, *P* <0.001), anxiety (β = 0.299, p<0.001), and suicide attempt (β = 0.151, *P* = 0.003). BE symptoms were positively associated with suicide attempt (β = 0.087, p = 0.011). BE symptoms and anxiety showed bidirectional association with each other (β = 0.018, p = 0.003) (Fig 1).

## Discussion

This study examined the relationship between BE symptoms and suicidality in patients with MDD. BE symptoms were associated with increased suicide attempts in patients with MDD and showed significant associations with suicide attempts even after adjusting for psychiatric comorbid conditions and trauma history.

BE symptoms were not uncommon in MDD; 17.38% of our subjects experienced BE symptoms, with 9.18% experiencing FBE. On the contrary, only 0.7% (six subjects) had comorbid eating disorders (anorexia nervosa or bulimia nervosa). The findings are consistent with recent studies that showed individuals with depression frequently experience disordered eating symptoms that do not fulfill the DSM criteria for anorexia nervosa or bulimia nervosa [21, 22]. Although BE behavior did not meet the full DSM criteria for eating disorder, it might represent underlying traits that raise suicide risk. In line with our findings, DSM-5 [23] newly includes binge eating disorder as an eating disorder subcategory. Individuals with binge eating disorder experience binge eating frequently without compensatory behavior. In a recent epidemiological study conducted in the United States, binge eating disorder was more common (lifetime prevalence 2.3%) than anorexia nervosa or bulimia nervosa, and was associated with various psychiatric comorbid conditions [24].

Although BE symptoms were not evaluated in association with depressive episodes, the MDD with BE group experienced increased appetite and weight gain more frequently during depression than the MDD without BE group. Previous studies also reported that atypical depression frequently co-occurs with bulimia nervosa [25, 26]. Atypical depression is usually associated with early age of onset, mood reactivity, which corroborates the findings of our study that the MDD with BE group were younger and had earlier age of onset. But, the MDD with BE group in our study did not show increased sleep more frequently than the MDD without BE group.

We additionally found that BE symptoms were associated with sexual trauma experience in MDD. Previous studies showed childhood trauma increases the risk of developing eating disorder by alterations in brain reward/ motivation pathways [27]. In particular, sexual trauma has been reported to increase the risk of BE/purging symptoms [28, 29]. The MDD with BE group showed higher rates of trauma regardless of the age of experience. People experiencing trauma engage in tension reduction behaviors to manage the after effects, the intensity of emotions, or the distressing aspects of the traumatic experience [30]. BE may be used to sooth, numb, or distract from the traumatic memory in the context of emotional eating in case of trauma experience [31]. In evaluating subjects with BE, trauma experience needs to be thoroughly examined.

Multivariate logistic regression analysis revealed that suicide attempts were associated with BE symptoms after adjusting for age, gender, income, and comorbidities, although the association did not reach the statistical significance (*P* = 0.022). It is notable that the MDD with FBE group did not show increased risk of suicide attempts compared to the MDD with ABE group. In terms of clinical characteristics including psychiatric comorbidity, no significant difference was detected between the MDD with ABE group and the MDD with FBE group. This finding might indicate that the frequency of BE symptoms did not differentiate the underlying traits associated with suicide attempt.

SEM showed sexual trauma increased BE symptoms and associated comorbid psychopathology, which eventually increased suicide risk in MDD. As previously discussed, BE may be used to sooth or numb oneself after a traumatic experience. At the same time, BE may reflect underlying affect dysregulation [32] and impulsivity [33, 34]. Biologically, it also might alternate tryptophan and serotonin [35] and lipid metabolism [36], which eventually may increase suicide risk.

The findings in this study should be interpreted in the context of how the study is designed. First, the CIDI interview was conducted by trained interviewers, who were not medical professionals. But, the validity and reliability of DSM-based diagnoses in the KECA, was fairly acceptable. Second, although loss of control was the essential part of the BE, it was not directly evaluated. However, the BE item in the CIDI has been widely used in previous studies [37–40]. A subjective loss of control is difficult to be evaluated and to be differentiated from the overeating [41]. Considering CIDI rather underdiagnosed eating disorder than overdiagnosed it [42], the BE defined in our study is less likely to be overestimated. Third, we did not evaluate detailed precipitating or associated factors of the BE experience. The BE can be associated with other behavioral changes such as stressful event or sleep deprivation [43]. We also did not evaluate how long the BE symptoms might persist, or how they might be temporally associated with depressed mood. We also cannot conclude any causal relationship from our study findings. As in a previous study conducted [44], suicide attempt might occur before BE symptoms. But BE tends to be a behavioral trait that persists [4]. Fourth, this study was conducted with a Korean population. Since depression symptomatology varies across ethnic groups and cultural background, further study with other ethnic groups is necessary to generalize the findings of our study.

In summary, BE symptoms were associated with suicide attempt in subjects with MDD. MDD with BE group had psychiatric comorbid conditions and sexual trauma history more frequently compared to MDD without BE group. Detailed evaluation on psychopathology including eating behavior and trauma history might lower suicide risk in MDD.
